# Icariin alleviates triptolide-induced testicular vacuolization via modulating germline ferroptosis and blood-testis barrier integrity

**DOI:** 10.3389/fcell.2026.1846734

**Published:** 2026-07-02

**Authors:** Lei He, Xiwen Yang, Tiantian Wu, Zhiran Li, Dan Zhao, Yangbo Fu, Yuting Chu, Jinglin Dong, Jiaxin Li, Qiuru Huang, Cong Shen, Chenyu Wang, Xinda Wang, Chen Qiao, Xiaorong Wang, Qingxia Meng, Jun Yu, Fei Sun

**Affiliations:** 1 Department of Urology, Nantong First People's Hospital, Southeast University Affiliated Nantong First People's Hospital, Nantong, China; 2 Institute of Reproductive Medicine, Jiangsu Province Key Laboratory in University for Inflammation and Molecular Drug Target, Medical School, Nantong University, Nantong, China; 3 School of Basic Medical Sciences, Key Laboratory of Fertility Preservation and Maintenance of Ministry of Education, Ningxia Medical University, Yinchuan, China; 4 State Key Laboratory of Reproductive Medicine and Offspring Health, Center for Reproduction and Genetics, The Affiliated Suzhou Hospital of Nanjing Medical University, Suzhou Municipal Hospital, Gusu School, Nanjing Medical University, Suzhou, China; 5 State Key Laboratory of Reproductive Medicine and Offspring Health, Department of Histology and Embryology, School of Basic Medical Sciences, Nanjing Medical University, Nanjing, China; 6 Department of Andrology, Nanjing Drum Tower Hospital, The Affiliated Hospital of Nanjing University Medical School, Nanjing, China; 7 Reproductive Medicine Center, The Fourth Affiliated Hospital of Jiangsu University, Zhenjiang, China; 8 Reproductive Sciences Institute, Jiangsu University, Zhenjiang, China; 9 Department of Clinical Pharmacy, Affiliated Hospital of Jiangsu University, Jiangsu University, Zhenjiang, China; 10 Clinical Research Center for Reproductive Genetics of Nantong University, Center for Reproductive Medicine, Affiliated Maternity and Child Health Care Hospital of Nantong University, Medical School, Nantong University, Nantong, China; 11 Center for Reproductive Medicine, Affiliated Maternity and Child Health Care Hospital of Nantong University, Nantong, China

**Keywords:** ferroptosis, icariin, spermatogenic niche, testicular vacuolization, triptolide

## Abstract

**Introduction:**

Environmental factors mediated testicular vacuolization injury is a prevalent occurrence, and its etiology and mitigation strategies have long remained inadequately elucidated.

**Methods:**

A testicular injury model was established using Triptolide (TP), with Icariin (ICA) employed as a rescue compound. Function and mechanism investigations primarily employed testicular coefficient and sperm concentration, H&E staining, immunofluorescence staining, and western blot analysis. Single-cell RNA-sequencing (scRNA-seq) predominantly examined the impacts of TP and ICA on various cell populations within the testis at a single-cell resolution.

**Results:**

We successfully established a TP-induced model of testicular injury and identified ICA as a protective agent in alleviating testicular vacuolization. Moreover, we delineated a comprehensive single-cell transcriptome profile of ICA in the repair of testicular injury, revealing the pivotal role of Sertoli-germline communications in the genesis of testicular vacuolization and the reparative process mediated by ICA. Furthermore, our investigation unveiled that ICA mitigated TP-induced damage to niche integrity through signatures associated with the blood-testis barrier (BTB), thereby averting substantial germ cell loss via the germline associated-ferroptosis signatures, potentially a key factor in the occurrence of testicular vacuolization injury. Additionally, we also identified ferroptosis-related molecules for testicular vacuolization injury.

**Discussion:**

We suggest that TP-induced testicular injury disrupts the spermatogenic microenvironment mediated by the BTB, leading to the formation of testicular vacuoles through the ferroptosis pathway in germ cells. Our findings offer fresh perspectives for ICA on mitigating this process.

## Introduction

Testicular injury, particularly testicular vacuolation, is a key etiological factor in male infertility, leading to focal disruptions in the spermatogenic microenvironment and consequent decline in sperm quantity and quality (Zhu et al., 2022; Chao et al., 2023; Zhang et al., 2024). Studies demonstrate that factors, such as environmental pollutant exposures and intake of medications, may result in testicular injuries, significantly impacting the functionality and structural integrity of the testicular spermatogenic microenvironment (Ma et al., 2019; Sharma et al., 2021; Zeng et al., 2023). Nevertheless, the etiology of testicular vacuolar injury and treatment strategies aimed at enhancing the local microenvironment largely remain enigmatic and require further elucidation.

A series of flavonoids have been extracted and identified from Epimedium (Mok et al., 2010; Zhou et al., 2015; Ding et al., 2025), Icariin (ICA) standing out as its primary active component, exhibiting a diverse array of pharmacological properties (Li et al., 2014; Fan et al., 2016). ICA has demonstrated antioxidant, anti-inflammatory, cardioprotective, hepatoprotective and antidepressant effects, rendering it a valuable therapeutic agent for conditions like cardiovascular diseases and tumors (Kong et al., 2015; Jia et al., 2019; Zhou et al., 2014; Zhang et al., 2017). Epimedium is frequently integrated into formulations for the treatment with male infertility (Chuang et al., 2021). Nevertheless, the exact mechanisms by which ICA alleviates testicular vacuolar injury and improves the testicular germline microenvironment remain ambiguous.

Triptolide (TP) has been validated to exhibit reproductive toxicity (Xi et al., 2017), primarily affecting sperm health. Treatment with TP markedly reduces the quantity and vitality of sperm in mice epididymis (Ma et al., 2015). Post TP treatment, male mice display significant shedding of germ cells, and various testicular injuries within the seminiferous tubules (Li J. et al., 2024). Recent study has indicated that prolonged intraperitoneal administration of TP (60 μg/kg) for 35 days effectively induces testicular vacuolization injury, establishing it as a pivotal model for investigating focal testicular damage (Yang et al., 2024). This study aims to further investigate the therapeutic impact of ICA on TP-induced testicular vacuolar injury and elucidate the regulatory mechanisms by which ICA repairs such injuries.

## Results

### Safety assessment of ICA in mice testes

To assess the testicular toxicity of ICA, we orally administered ICA to mice for 35 consecutive days (Supplementary Figure S1A). Initially, we found that ICA did not impact weight size and testicular coefficient (Testis/Body weight) in both low (50 mg/kg) and high (150 mg/kg) concentrations of ICA groups when compared with control group (Supplementary Figures S1B,C). Computer-assisted sperm analysis (CASA) also demonstrated that ICA did not influence the sperm concentration in mice epididymis (Supplementary Figures S1D,E). Subsequently, histological examinations using hematoxylin and eosin (H&E) staining revealed that treatment with ICA did not result in significant morphological alterations: the structure of the seminiferous tubules remained intact, the seminiferous epithelium showed no signs of damage, and the different levels of spermatogenic cells were neatly arranged (Supplementary Figure S1F).

### The protective effects of ICA on TP-induced testicular injury

**FIGURE 1:**
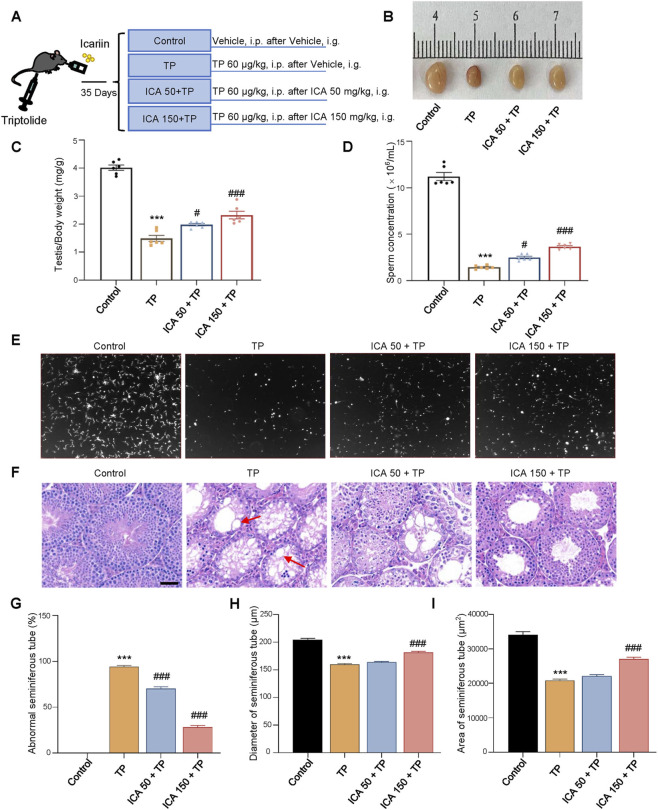
Protective effects of ICA against testicular damages induced by TP in mice. **(A)** Illustration of animal treatments for ICA and TP. **(B)** Testicular morphology and size in Control, TP, ICA50 +TP, and ICA150 +TP groups. **(C)** Testis/Body weight (mg/g) in Control, TP, ICA50 +TP, and ICA150 +TP groups; n = 6. **(D)** Sperm concentration in Control, TP, ICA50 +TP, and ICA150 +TP groups; n = 6. **(E)** Sperm morphology under CASA in Control, TP, ICA50 +TP, and ICA150 +TP groups. **(F)** HE stainings of testicular sections in Control, TP, ICA50 +TP, and ICA150 +TP groups. The red arrows mark the representative testicular vacuolization structures. **(G)** Ratio of abnormal seminiferous tube in Control, TP, ICA50 +TP, and ICA150 +TP groups; n = 100. **(H)** Diameter of seminiferous tube in Control, TP, ICA50 +TP, and ICA150 +TP groups; n = 50. **(I)** Area of seminiferous tube in Control, TP, ICA50 +TP, and ICA150 +TP groups; n = 50. Statistically compared with the Control group (***p < 0.001) and the TP group (#p < 0.05; ###p < 0.001); Scale bar: 50 μm.

We next employed male mice to establish a TP-induced testicular injury model, with treatment using ICA and subsequent evaluation of relevant parameters after 35 days of continuous administration (Figure 1A). Findings indicated that TP exposure led to significant decreases in both testicular size and testicular coefficient, and administration of ICA resulted in dose-dependent recovery effects (Figures 1B,C). CASA results also indicated that TP exposure caused a notable reduction in epididymal sperm concentration, while partial rises in sperm concentration were observed in the ICA + TP groups (Figures 1D,E). H&E staining revealed that, compared with the control group, TP exposure induced testicular injury characterized by disordered germ cell arrangement, the presence of vacuolar structures of varying sizes in the seminiferous tubules, shedding of germ cells in most tubules, and reduced sperm production efficiency, while treatment with ICA significantly alleviated testicular injuries (Figure 1F). Statistical analysis for the ratio, diameter, and area of abnormal seminiferous tube further demonstrated the protective role of ICA against TP-induced testicular injury (Figures 1G–I). Collectively, these results indicate that ICA confers protective effects against testicular injuries and decreased sperm concentration induced by TP.

### Single-cell transcriptome landscape of ICA for testicular injury repair

**FIGURE 2:**
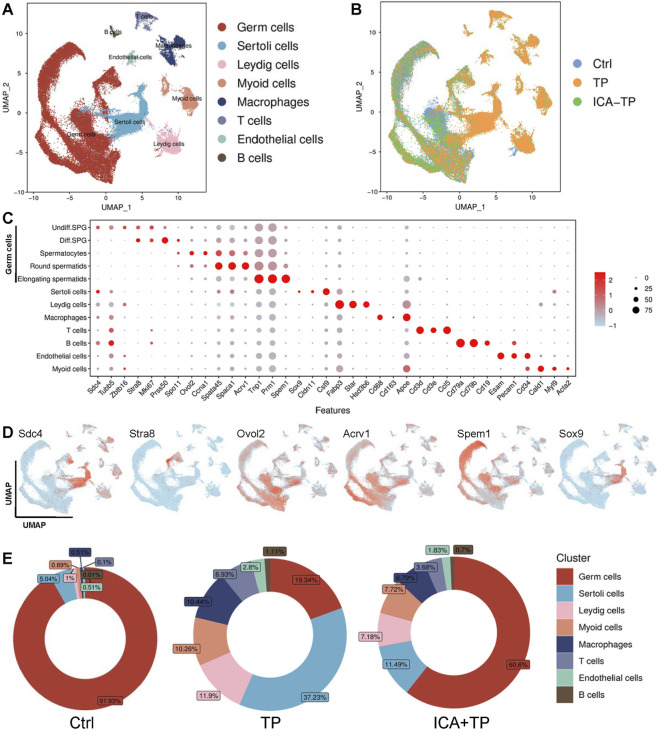
Single-cell transcriptomics for ICA against TP-induced testicular damages. **(A)** UMAP visualization illustrating testicular cell populations in mice. **(B)** UMAP visualization of testicular cell populations in Control, TP, and ICA + TP groups. **(C)** Dotplot for marker genes within each testicular cell population. **(D)** UMAP visualizations for representative marker genes. **(E)** Fraction of testicular cell populations in Control, TP, and ICA + TP groups.

To further investigate the role of ICA for the testicular injury repair, we characterized cells using single-cell RNA sequencing (scRNA-seq) from freshly dissected testes of control, TP, and ICA (150 mg/kg)+TP (named as ‘ICA + TP’) groups. In total, we acquired 66,183 cells, uniformly distributed in the Uniform Manifold Approximation and Projection (UMAP) visualization. Cells of excellent quality underwent sequencing, revealing a median of 3,353 unique molecular identifiers (UMIs) and a median of 1,878 genes detected per cell. These cells were distinctly categorized into eight major cell populations (Figure 2A). We subsequently observed testicular cells *via* groups using UMAP plot (Figure 2B). Overall, TP exposure resulted in significant changes in the number of several cell populations, while the number of these cell populations in the ICA + TP group was restored (Supplementary Figure S2A). To annotate these clusters precisely, we cross-referenced the expression of well-characterized marker genes, using Dotplot view to confirm their identities (Figure 2C; Supplementary Figure SI), including germ cells (Udiff.SPG, Diff.SPG, Spermatocytes, Round spermatids, Elongating spermatids), Sertoli cells, Leydig cells, Macrophages, T cells, B cells, Endothelial cells and Myoid cells. Representative marker genes were further mapped onto UMAP plots to visualize their distribution (Figure 2D; Supplementary Figure S2B). The top five most abundantly expressed genes in major cell clusters were shown to further understand the expression characteristics of various cell populations in testes (Supplementary Figure S2C). It is noteworthy that both the proportion and absolute count of germ cells exhibited a significant decrease, whereas Sertoli cells showed a marked increase following TP exposure (Figure 2E; Supplementary Figures S2D,E). These changes were substantially mitigated by ICA, suggesting that ICA can effectively restore core niche components damaged by TP exposure, thereby alleviating testicular injury.

### ICA ameliorates TP-induced integrity damage of blood-testis barrier (BTB)

**FIGURE 3:**
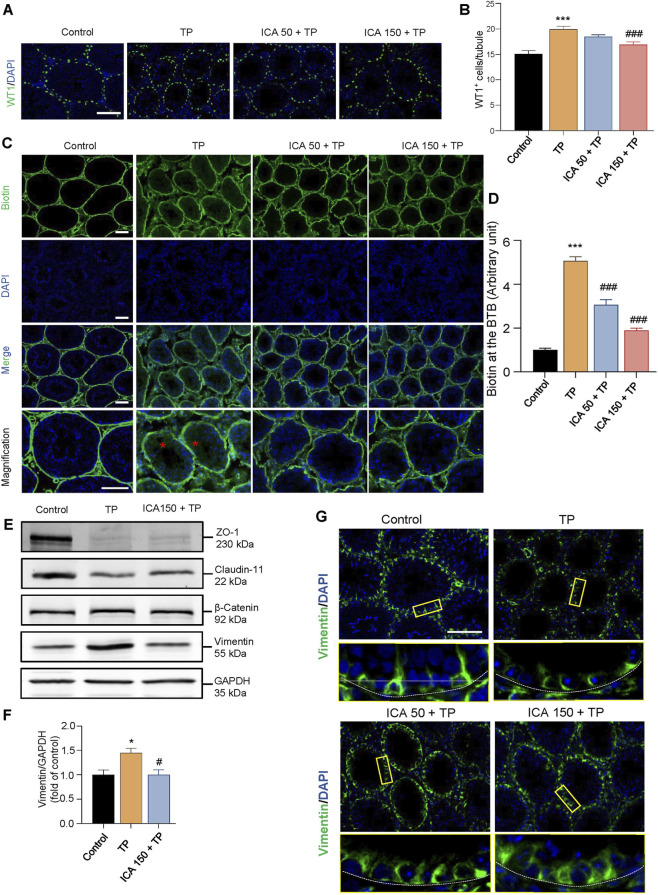
Protective effects of ICA on the BTB integrity against TP-induced testicular damages. **(A)** Immunofluorescence staining of WT1 in testicular sections of each group. **(B)** Quantification of WT1 positive Sertoli cells per tubule in each group. **(C)** Assessment of BTB Integrity: In the control testes, the biotin-Alexa Fluor 488 signal was localized adjacent to the basement membrane and effectively prevented the diffusion of membrane-impermeable biotin across the barrier. After TP treatment, the biotin-Alexa Fluor 488 signal penetrated the BTB and entered the seminiferous tubules (* indicated), indicating BTB disruption. ICA significantly mitigated TP-induced damages to BTB integrity. **(D)** Biotin at the BTB (Arbitrary unit) in Control, TP, ICA50 +TP, and ICA150 +TP groups. **(E)** Western blot analysis of expression levels of ZO-1, Claudin-11, β-Catenin, Vimentin and GAPDH proteins in Control, TP, and ICA150 +TP testes. **(F)** Statistical analysis of Vimentin protein levels in Control, TP, and ICA150 +TP testes. **(G)** Immunofluorescence staining of Vimentin in testicular sections of each group. Statistically compared with the Control group (*p < 0.05; ***p < 0.001) and the TP group (#p < 0.05; ###p < 0.001); Scale bar: 100 μm.

We next performed immunofluorescence staining with WT1 transcription factor (WT1), discovering that TP exposure did not decrease but instead increased the number of Sertoli cells, and the abnormal increase in the number of Sertoli cells could be reversed under the protective effect of ICA (Figures 3A,B). To further clarify the effect of ICA on the BTB induced by TP, we analyzed the BTB integrity using a biotin tracer (Figures 3C,D): TP exposure destroyed BTB integrity, allowing biotin to enter into the seminiferous tubules; treatment with ICA significantly ameliorated TP-induced BTB integrity damages, and the amount of biotin entering the seminiferous tubules was significantly reduced. Next, Western blot was used to detect the expression levels of BTB associated proteins. Compared with the control group, TP exposure decreased the expression levels of ZO-1 and Claudin-11 proteins, while the expression level of β-Catenin remained unchanged in testes (Figure 3E; Supplementary Figure S3A). Moreover, TP exposure could increase the expression level of Vimentin protein, and ICA could recover its expression level (Figures 3E,F).

The top extension of Sertoli cells contacts with the germ cells, guiding them to migrate towards the lumen of the seminiferous tubules (Wang et al., 2024; Jin et al., 2025). After TP exposure, we also observed the loss of apical extension of Vimentin signature in Sertoli cells (Figure 3G). And the distribution of tight junction protein ZO-1, which is no longer tightly distributed on the basement membrane, but diffusely distributed (Supplementary Figure S3B). By staining with α-Tubulin, it could be observed that microtubules present a “track like” structure, arranged perpendicular to the basement membrane and extending throughout the entire seminiferous epithelium in control testes, while many orbital like structures were disordered, and microtubule structures almost collapsed and covered the basement membrane after TP exposure (Supplementary Figure S3C). Importantly, we uncovered that ICA could partially recover the distribution defects of skeleton proteins and tight junction proteins induced by TP exposure (Figure 3G; Supplementary Figures S3B,C). The above results indicate that ICA can protect the integrity of BTB structure and function by alleviating the expression and distribution of skeletal proteins and tight junction proteins.

### ICA ameliorates TP-induced spermatogenesis disorder

**FIGURE 4:**
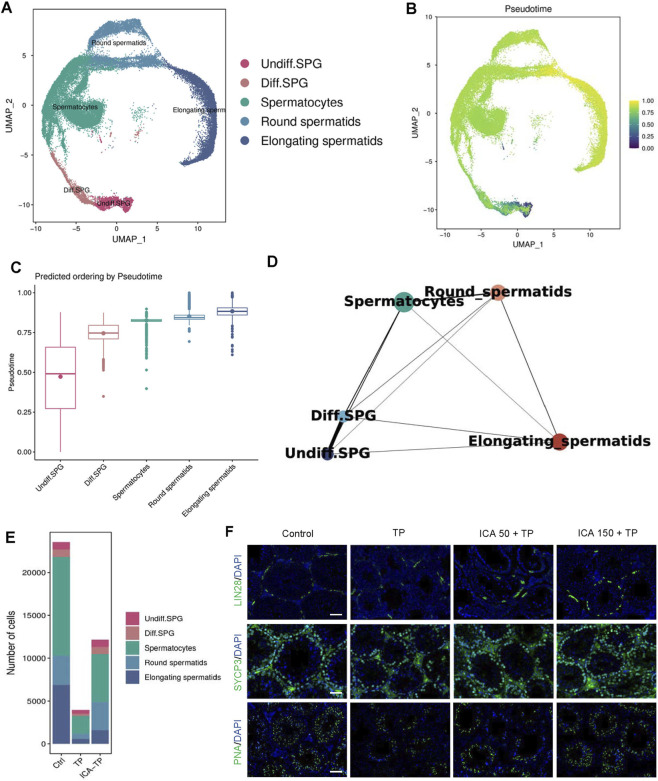
Features of germ cells for ICA against TP-induced testicular damages. **(A)** UMAP visualization of germ cells at different stages. **(B)** UMAP visualization of germ cell populations *via* pseudotime analysis based on PAGA algorithm. **(C)** Predicted ordering by pseudotime for different stages of germ cells. **(D)** The PAGA network diagram to determine the strength of differentiation inheritance relationships among germ cells. **(E)** The number of different stage germ cells in Control, TP, and ICA + TP groups. **(F)** Stainings of LIN28, SYCP3 and PNA in testicular sections of each group. Scale bar: 50 μm.

To explore the differentiation complexity of germ cell populations, we re-clustered the germline populations and visualized in the UMAP plot, uncovering that TP exposure resulted in significant decrease of germ cells and ICA restore these changes (Figure 4A; Supplementary Figure S4A). The top five most abundantly expressed genes in each stage of germ cell population were shown with heatmap (Supplementary Figure S4B). We thereby employed Partition-based graph abstraction (PAGA) algorithm for pseudotime analysis of germ cell populations, and mapped the pseudotime values onto the UMAP plot of germ cell populations (Figure 4B). Pseudotime trajectory analysis re-constructed an inferred transitional trajectories of germline populations at different stages, which were consistent with the trend of germ cell differentiation (Figure 4C). We also performed a PAGA network diagram to determine the strength of differentiation inheritance relationships among germline populations at different stages (Figure 4D).

scRNA-seq also revealed that all stages of germline populations were decreased after TP exposure, and treatment with ICA could alleviate the trend of cell reduction (Figure 4E). To further investigate the protective effects of ICA on spermatogenesis disruption induced by TP, we performed stainings with germline markers, including LIN28 for spermatogonia, SYCP3 for spermatocytes, and PNA for spermatids in the testicular seminiferous tubules post-treatments (Figure 4F). Upon statistical analysis (Supplementary Figures S4C–E), we observed significant decreases in the number of germ cells at various developmental stages due to TP exposure. Under the protective role of ICA, there were increases in the quantity of germ cells *via* dose-dependent effects (Supplementary Figures S4C–E). These findings indicate that ICA can alleviate the testicular damage to germ cells caused by TP at different developmental stages in mice.

### ICA ameliorates TP-induced testicular vacuolization injury *via* GPX4-mediated ferroptosis

**FIGURE 5:**
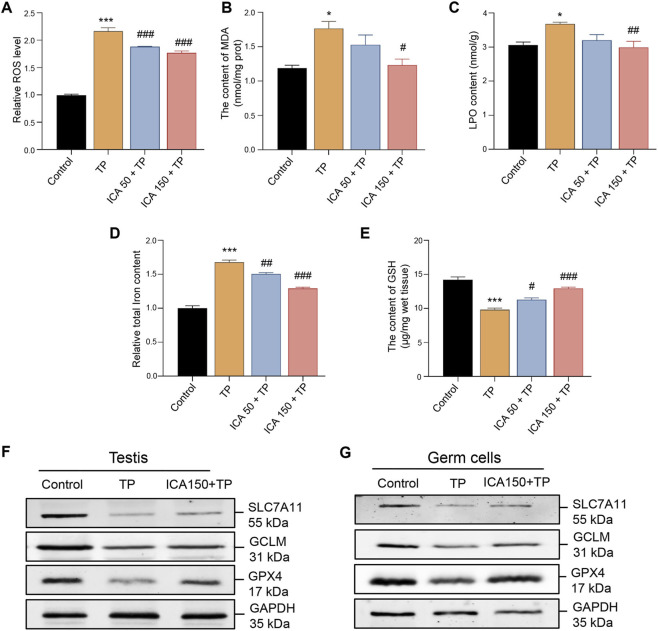
ICA alleviates TP-induced testicular oxidative stress damage. **(A)** Relative ROS level in each group of mice testes. **(B)** MDA content in each group of mice testes. **(C)** LPO content in each group of mice testes. **(D)** Relative total Iron content in each group of mice testes. **(E)** GSH content in each group of mice testes. **(F)** Western blot analysis of SLC7A11, GCLM, GPX4 and GAPDH protein levels in Control, TP, and ICA150 +TP testes. **(G)** Western blot analysis of SLC7A11, GCLM, GPX4 and GAPDH protein levels in isolated germ cells derived from the testes of Control, TP, and ICA150 +TP groups. *p < 0.05, **p < 0.01, ***p < 0.001 compared to the Control group; #p < 0.05, ##p < 0.01, ###p < 0.001 compared to the TP group.

Previous study has indicated that oxidative stress contributed to testicular damage (Zangene et al., 2024). Therefore, we assessed oxidative stress related indicators in mice testes post-treatments. Compared to the control group, TP exposure exhibited significant increases in ROS, malondialdehyde (MDA), lipid peroxidation (LPO) and total iron levels, and dramatic decrease in glutathione (GSH) content (Figures 5A–E). Following pretreatment with ICA and subsequent TP administration, ROS, MDA, LPO, total iron and GSH levels demonstrated varying degrees of recoveries in mice testes (Figures 5A–E), indicating that ICA could attenuate ferroptosis-associated damage. Moreover, we observed dramatic decreases in the protein expression levels of GPX4, SLC7A11 and GCLM in TP-treated testicular tissues and isolated germ cells (Figures 5F,G). Furthermore, under the combined influence of ICA and TP, we found that ICA can improve TP-induced ferroptosis approach by restoring the expression levels of GPX4 in both testicular tissues and isolated germ cells (Figures 5F,G), indicating that ICA can protect against TP-induced testicular ferroptosis by up-regulating GPX4 protein.

**FIGURE 6:**
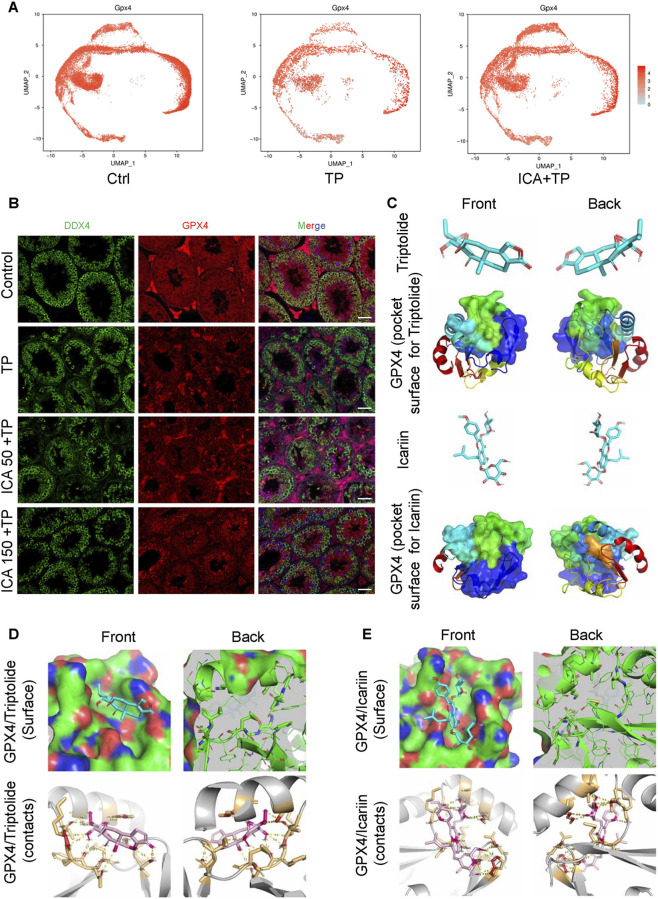
ICA and TP regulate testicular damages through GPX4. **(A)** UMAP visualizations of Gpx4 expression patterns in germline populations of Control, TP, and ICA + TP groups. **(B)** Immunofluorescence stainings of DDX4 (green) and GPX4 (red) in testicular sections of each group. Scale bar: 50 μm. **(C)** Three-dimensional structure of TP and ICA, and pocket surface of GPX4 protein for TP and ICA. **(D)** Key residues at GPX4/TP surface and interaction sites. **(E)** Key residues at GPX4/ICA surface and interaction sites.

By overview the expression pattern of GPX4 in germ cell populations *via* UMAP plot, we found that TP exposure induced widespread reduction of GPX4 expression in germ cell populations, while ICA could effectively restore its expression (Figure 6A). Fluorescence staining also verified that GPX4 protein co-localized with the germ cell marker DDX4, and GPX4 protein in germ cells was significantly reduced after TP exposure, and this decreased expression pattern could be restored by ICA treatment (Figure 6B). Furthermore, the three-dimensional structures of TP and ICA were visualized, and potential binding pockets of GPX4 protein for TP and ICA were analyzed (Figure 6C). Interactions between GPX4 and TP (or ICA) were conducted using CB-Dock2 (Figures 6D,E). The key residues at the interface of GPX4 and TP (or ICA) were visualized (Supplementary Tables S2, S3). The predicted interactions between TP/ICA and GPX4 suggest potential binding modes, but these findings still require experimental validation to confirm direct binding or functional regulation. Taken together, these data show that testicular vacuolization injury is associated with reduced GPX4 levels, a hallmark of ferroptosis, and that ICA treatment restores GPX4 expression while ameliorating the injury.

### scRNA-seq reveals novel targets for testicular vacuolization injury

**FIGURE 7:**
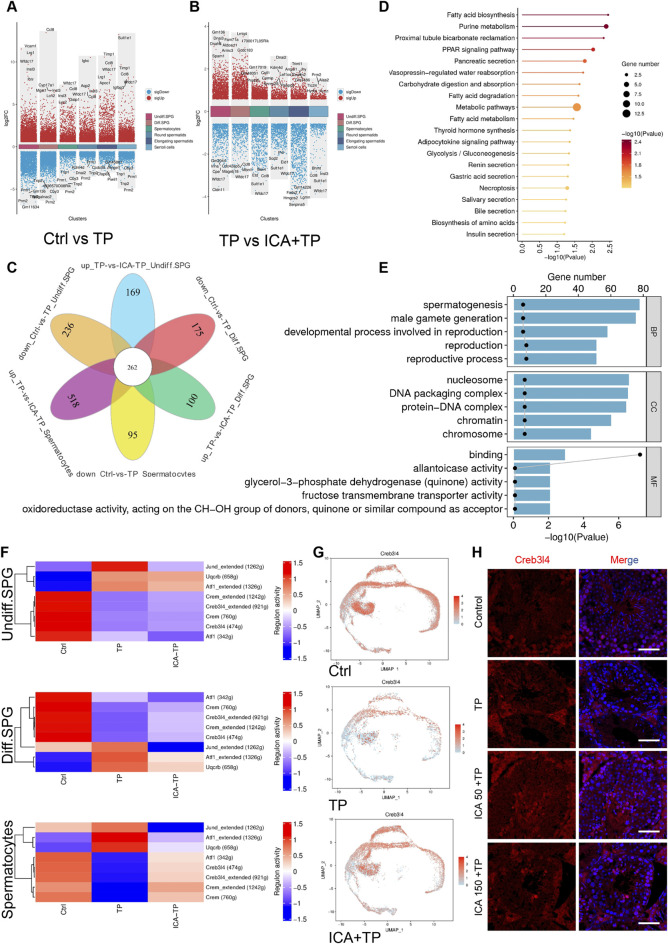
Identification of pivotal factors within germline populations regarding the amelioration of testicular damages by ICA. **(A)** Analysis of DEGs within primary testicular niche populations between the Control and TP groups. **(B)** DEGs analysis within primary testicular niche populations between the TP and ICA + TP groups. **(C)** Venn diagram illustrating the core DEGs that are downregulated between the Control and TP groups and upregulated between the TP and ICA + TP groups across Undiff.SPG, Diff.SPG and Spermatocytes populations. **(D)** KEGG analysis of the common core DEGs. **(E)** GO analysis of the common core DEGs. **(F)** Heatmap displaying regulons targeting Gpx4 in Undiff.SPG, Diff.SPG and Spermatocytes populations of the Control, TP, and ICA + TP groups. **(G)** UMAP visualizations of Creb3l4 expression patterns in germline populations of the Control, TP, and ICA + TP groups. **(H)** FISH analysis of Creb3l4 expression patterns in testicular sections of each group. Scale bar: 50 μm.

Given the limited knowledge surrounding testicular vacuolization injury, our subsequent investigation aimed to identify potential novel targets involved in the regulation of these conditions by analyzing single-cell transcriptomic profiles. Differentially expressed genes (DEGs) were identified for the comparisons of control and TP groups (Figure 7A; Supplementary Table S4), and the comparisons of TP and ICA + TP groups (Figure 7B; Supplementary Table S5) in major niche cellular populations. Due to the fact that testicular vacuoles may mainly occur in pre-meiotic and meiotic cells, we therefore screened for novel targets with similar expressed alterations (e.g., GPX4) post-treatments. Kyoto Encyclopedia of Genes and Genomes (KEGG) revealed that the above identified DGEs mainly participated in cell cycle, ribosome, spliceosome and multiple metabolic processes in Udiff.SPG, Diff.SPG, Spermatocytes sub-clusters post-treatments (Supplementary Figure S5). Among these germline populations, we further uncovered 206 core DEGs downregulated after TP exposure, and upregulated in ICA + TP group when compared with TP exposure alone (Figure 7C). KEGG enrichment also indicated that these core DEGs were involved in multiple metabolic pathways, fatty acid biosynthesis and degradation, biosynthesis of amino acids, necroptosis, and so on (Figure 7D). Additionally, Gene Ontology (GO) enrichment analysis indicated that these core DEGs participated in spermatogenesis and reproductive process (Figure 7E).

To further refine our list of candidate targets, we subsequently constructed the transcription factors (TFs) regulatory network module *via* Regulon to predict TFs and their corresponding target genes. Within this context, we identified a series of TFs targeted Gpx4 in Udiff.SPG, Diff.SPG, Spermatocytes sub-clusters, respectively (Figure 7F). Importantly, among these TFs, Creb3l4 was the only identified DEGs with the similar expression pattern of GPX4 in germline populations of control, TP, ICA + TP groups (Figure 7G). We subsequently utilized a fluorescence *in situ* hybridization (FISH) assay to illustrate the predominant localization of Creb3l4 within germ cells (Figure 7H). Following exposure to TP, a significant reduction in its expression was observed, which could be effectively restored through ICA treatment (Figure 7H). Taken together, our results identify Creb3l4 as a potential novel target for testicular vacuolization injury.

## Discussion

Due to a multitude of factors such as environmental pollution, increased work pressure, and unhealthy lifestyle habits, human reproductive capacity has significantly declined (Segal and Giudice, 2019; Wang et al., 2023; Khodadadi et al., 2025). Among these, environmentally mediated testicular vacuolation injury may further lead to focal testicular damage. However, the regulatory mechanisms of testicular vacuolation injury have been consistently underestimated by clinical and basic research, often resulting in cases of infertility being categorized as idiopathic male infertility. This study utilized the previously validated TP-induced testicular vacuolization injury model to further investigate the role of ICA in alleviating testicular vacuolation injury (Yang et al., 2024). Our key findings include: a) ICA serves as a crucial protective agent against testicular vacuolation injury; b) ICA can repair BTB integrity damage by modulating the expression levels and patterns of BTB-related molecules, thereby providing a new testicualr niche for spermatogenesis; c) ICA can repair testicular vacuolation injury by inhibiting the ferroptosis pathway in germ cells; d) Further utilization of scRNA-seq indicates that Creb3l4 may be a novel target for testicular vacuolization injury.

Epimedium, as a representative traditional Chinese medicinal herb, was first documented in the classic Chinese medical work “Shen Nong Ben Cao Jing” and has a history of over two thousand years (Wang et al., 2021). ICA, a natural multifunctional flavonoid compound extracted from the stem and leaves of Epimedium, serves as the primary active ingredient of Epimedium extract, possessing various pharmacological activities such as anti-inflammatory, anti-aging, anti-tumor, antidepressant, bone metabolism promotion, and immune-protective effects (Xu et al., 2010; Xu et al., 2020; Wang et al., 2018; Cao et al., 2019; Li et al., 2021). Environmental pollution and other factors are significant contributors to testicular injuries, leading to apoptosis of male germ cells, subsequently causing the decrease in male fertility, characterized by oligozoospermia and azoospermia (Pohl et al., 2021; Li X. et al., 2024). Previous studies have indicated that ICA could enhance antioxidant capacity, ameliorate testicular damage and germ cell apoptosis, thereby aiding in the restoration of male fertility (Sun et al., 2019; Ni et al., 2020). The administration of ICA for a duration of 35 days can effectively reverse the substantial decline in antioxidant enzyme activities and the increase in MDA levels triggered by nicotine in mice (Ni et al., 2020). Additionally, our study also reveals that ICA can selectively regulate the ferroptosis pathway in germ cells of mice testes to inhibit cell death, primarily by restoring the oxidative stress balance. Taken together, ICA not only mediates the protective effect on reproductive function by regulating ferroptosis, but also protects reproductive function through multiple mechanisms including activating the PI3K/AKT pathway, activating ERα/Nrf2, and regulating the glycolytic pathway (Sánchez-Gutiérrez et al., 2024; Li F. et al., 2024). These evidence indicate that ICA can improve testicular germline niche damages by alleviating oxidative stress imbalance.

Ferroptosis is a novel iron-dependent programmed cell death approach caused by the excessive accumulation of LPO (Stockwell et al., 2017; Jaeschke and Ramachandran, 2024). Current study indicates that iron overload and oxidative stress are significant factors triggering cellular ferroptosis (Wan et al., 2019; Li S. et al., 2024). This study primarily reveals that TP-induced testicular injuries can alter the expression levels and patterns of BTB-associated proteins, disrupting the germline niche in mice testes. Further investigation shows that TP can target the activation of the germline ferroptosis by influencing the expression of GPX4, which is considered as a crucial factor leading to testicular vacuolization injury. To further distinguish ferroptosis from other cell death pathways, future studies will include the use of ferroptosis-specific inhibitors and other cell death inhibitors. Moreover, through protein-compound docking and interaction interface analysis (Senior et al., 2020; Liu et al., 2022), we found that TP and ICA both possess the ability to bind to GPX4 protein, thereby exerting their functions. Although most ferroptosis-related markers were measured in whole testis, our isolated germ cell data and immunofluorescence co-localization support a germline-specific contribution. While our results show consistent changes in GPX4 expression and its recovery by ICA, these findings do not prove causality. Future GPX4 modulation/rescue experiments are required to establish GPX4 as a functional mediator.

Unexpectedly, we discover that Creb3l4 may be a novel regulator of testicular vacuolization injury through scRNA-seq. The altered expression patterns of Creb3l4 in response to TP alone and in combination with ICA are similar to that of GPX4. Moreover, we have identified Creb3l4 as the only TF exhibiting specific expression pattern and TF activity across the different treatment groups, with GPX4 being its downstream regulatory target. Creb3l4, a member of the CREB/ATF transcription factor family, is recognized for its regulation of cell proliferation, differentiation, and apoptosis, showing a high degree of similarity in primary and secondary protein structures between mice and humans (Velpula et al., 2012). Previous study indicated that *Creb3l4-deficient* mice contained all of the developmental stages and only exhibited increased apoptosis of germ cells (Adham et al., 2005). Based on the published literature and our major findings, it suggests that Creb3l4 is not a necessary gene for complete spermatogenic arrest, but rather an important regulatory factor in focal testicular damage.

The cellular effects of TP and ICA may be broad and multi-targeted. This study notably focuses on the role of ICA in core cellular clusters within the testicular niche induced by TP, while further exploration is necessary to explore whether TP can impact the survival of other testicular cell types and the reparative effects of ICA on damage to these cells. Future studies will continue to utilize single-cell transcriptome data to delve deeper into the cellular effects and targets of TP and ICA in various testicular cell types.

In summary, this study systematically assesses the impact of ICA on testicular vacuolization injury, elucidating the molecular mechanism by which ICA regulates signatures maintaining the BTB integrity in Sertoli cells and inhibits the ferroptosis pathway in germ cells. Therefore, we introduces the innovative concept that ICA ameliorates the testicular spermatogenic niche through a dual-cell, dual-pathway approach. These findings offer substantial backing for deeper comprehension of regulatory targets for ICA in testicular vacuolization injury induced by environmental factors, and present novel avenues for investigating the additional benefits of traditional Chinese medicine in focal testicular damage.

## Materials and methods

### Animal source and grouping scheme

The experiments utilized 8-week-old male C57BL/6J mice housed in a SPF level animal facility with a room temperature of 25 °C and a 12-h light/dark cycle. All animals in the experiment had *ad libitum* access to food and water.

TP (purity >98%) was purchased from Sichuan Weikeqi Biotechnology Co., Ltd., and ICA (purity >95%) was purchased from Shanghai Luoen Chemical Technology Co., Ltd. To assess the safety of ICA, mice were divided into three groups: (1) Control group, gavage with physiological saline; (2) ICA 50 mg/kg group, gavage with 50 mg/kg of ICA; (3) ICA 150 mg/kg group, gavage with 150 mg/kg of ICA. To investigate the protective effect of ICA on TP-induced testicular injuries, mice were divided into four groups: (1) Control group, gavage with physiological saline followed by intraperitoneal injection of physiological saline containing 1% DMSO; (2) TP group, gavage with physiological saline followed by intraperitoneal injection of 60 μg/kg TP; (3) ICA 50 mg/kg + TP group, gavage with 50 mg/kg of ICA followed by intraperitoneal injection of 60 μg/kg TP; (4) ICA 150 mg/kg + TP group, gavage with 150 mg/kg of ICA followed by intraperitoneal injection of 60 μg/kg TP. Tissues were collected after 35 consecutive days of administration.

### Testicular coefficient and sperm concentration

The mice as well as their testes were weighed, and the testicular weight index was calculated by dividing the testicular weight (mg) by the body weight (g). The caudal epididymides were removed and incubated in Tyrode’s solution (#T1421, Solarbio, Beijing, China) at 37 °C for 15 min to facilitate the release of sperm through gentle shaking. Following this, the sperm suspension was placed on a glass slide, and the sperm concentration was assessed using a CASA system (Hamilton-Thorne, Beverly, MA, United States of America).

### H&E staining

Tissues were fixed in modified Davidson’s fluid (MDF) for at least 48 h, dehydrated through a series of ethanol solutions of varying concentrations, and then embedded in paraffin (39,601,095, Leica, Germany). The tissues were sectioned at 5 μm thickness. These sections were deparaffinized in xylene for 20 min, followed by rehydration with a gradient of ethanol solutions at concentrations of 100%, 90%, 80%, and 70%. Subsequently, the sections were stained with hematoxylin and eosin (G1120, Solarbio, China) for 5 min and 2 min, respectively. Imaging was performed using a digital scanning microscope (Tissue Gnostics, Austria).

### Immunofluorescence staining

The dewaxed paraffin sections were treated with a citrate antigen retrieval solution (P0083, Beyotime, China), heated to boiling, and maintained at this temperature for 10 min. Subsequently, the slices were permeabilized with 0.1% Triton X-100 (ST1722, Beyotime, China) at room temperature for 30 min. After washing with PBS, the samples were blocked with 5% bovine serum albumin (BSA) at room temperature for 1 h. The sections were then incubated with primary antibodies (Supplementary Table S6) at 4 °C overnight. After washing, the tissues were incubated with secondary antibodies labeled with A488, Cy3, or A647 (Jackson ImmunoResearch Laboratories) for 1 h. Cell nuclei were visualized by staining with DAPI (C1005, Beyotime, China) for 20 min. Finally, the sections were observed under a fluorescence microscope (Zeiss, Germany).

### Biotin tracer assay

The EZ-Link™ Sulfo-NHS-LC-Biotin tracer (A39258, Thermo Fisher Scientific, United States of America) was dissolved in PBS at a concentration of 10 mg/mL and utilized to assess the BTB integrity in the testes. Mice were anesthetized, and 30 μL of the Biotin working solution was injected beneath the tunica albuginea of the testis. Approximately 30 min post-injection, the testes were dissected and sliced into 7-μm-thick cryosections. Once sectioning was completed, the sections were fixed with 4% paraformaldehyde (PFA) for 15 min, blocked with 5% BSA for 1 h, washed three times with PBS, and then incubated with Alexa Fluor™488-streptavidin (#S32354, Thermo Fisher Scientific, United States of America) for 1 h. Following this incubation, the sections were washed three times with PBS and stained with DAPI for 20 min. Imaging was carried out using a fluorescence microscope (Zeiss, Germany).

### Western blot

Proteins that underwent various treatments were extracted using RIPA lysis buffer (P0013B, Beyotime, China) supplemented with a protease and phosphatase inhibitor cocktail (P1045, Beyotime, China), and 1% phenylmethanesulfonylfluoride (PMSF, ST506, Beyotime, China). Following the experimental procedure, proteins were separated *via* 10% or 12% sodium dodecyl sulfate-polyacrylamide gel electrophoresis (SDS-PAGE) and then transferred onto nitrocellulose membranes (F619512-0001, Sangon Biotech, China). The membranes were blocked with 5% skimmed milk at room temperature for 1 h, followed by overnight incubation at 4 °C with specific primary antibodies (Supplementary Table S6). After a 15-min wash with TBST buffer, the membranes were exposed to anti-IgG (Alexa Fluor® 680, ab175773, Abcam, United States of America) for 1 h, washed again with TBST, and scanned using an Amersham Typhoon 5 Biomolecular Imager system (GE Healthcare Lifescience, UK).

### Ferroptosis related assays

Ferroptosis associated indicators were assessed through the measurement of total iron content (BC5315, Solarbio, China), LPO (BC5245, Solarbio, China), GSH (BC1175, Solarbio, China), and MDA (BC0025, Solarbio, China) levels. Testes tissues from distinct groups were obtained and lysed employing an ultrasonic homogenizer at 10,000 g for 10 min at 4 °C.In accordance with the manufacturer’s instructions, the resultant supernatants and detection reagents were transferred to 96-well plates and thoroughly mixed. The absorbance values (Iron: λ_510nm_; LPO: λ_532nm_; GSH: λ_412nm_; MDA: λ_532nm_) were determined using the FlexStation 3 Multi-Mode Microplate Reader (Molecular Devices, United States of America).

To detect ROS level, testicular tissues were obtained, and added to 500 μL of PBS, thoroughly ground on ice to prepare the single-cell suspension. Subsequently, DCFH-DA (S0033, Beyotime, China) was diluted in PBS at a ratio of 1:1,000 to achieve a final concentration of 10 μM. The single-cell suspension was centrifuged at 1,000 rpm for 5 min, the supernatant was discarded, and 500 μL of pre-prepared probe solution was added after resuspending the cells thoroughly. The cells were then incubated on ice under light-avoiding conditions for 1 h. Following incubation, the cells were washed for three times with PBS, and 100 μL of each sample was added to the wells, and fluorescence intensity was measured using an excitation wavelength of 488 nm and an emission wavelength of 525 nm *via* FlexStation 3 Multi-Mode Microplate Reader (Molecular Devices, United States of America).

### Single-cell suspensions, library preparation, sequencing and data processing

Freshly dissected testes were used to profile cells from six independent samples. The cell number and viability of single-cell suspensions were assessed using Trypan Blue staining. A Chromium instrument (10X Genomics, United States of America) was used to carry out the single-cell RNA-sequencing. A Chromium Single Cell 3′ Library and Gel Bead Kit v3 (10X Genomics) was used to process the samples. Gene *Denovo* Biotechnology Co., Ltd. (Guangzhou, China) then sequenced the gene expression libraries using a NovaSeq 6,000 sequencer (Illumina, United States of America).

The raw BCL files were converted to FASTQ files, aligned and the counts quantified using Cell Ranger software (version 6.1.0; 10X Genomics). Reads with low-quality barcodes were discarded, and UMIs were filtered out and mapped to the reference genome (gencode_release26M_GRCm39). Those reads that mapped uniquely to the transcriptome and intersected an exon by at least 50% were considered for UMI counting. Prior to quantification, the UMI sequences were corrected for sequencing errors, and the EmptyDrops method was used to identify valid barcodes (Lun et al., 2019). Cell barcode calling and UMI counting produced the cell by gene matrices. For the downstream analysis of each sample, the cell-by-gene matrices were imported individually into Seurat version 4.1.3 (Butler et al., 2018). Those cells having an unusually high number of UMIs (≥71,000) or mitochondrial gene percent (≥20%) were discarded. We also excluded cells with less than 240 or more than 8,500 detected genes. Additionally, doublet gel beads in emulsion (GEMs) were also filtered out using DoubletFinder (v2.0.3) (McGinnis et al., 2019). UMAP plot was generated to visualize the cell clusters. Genes that were upregulated and enriched were identified as significant based on the criterion of ln (fold change) greater than 0.25 and a p value below 0.01. DEGs were considered significant with a fold change more than 1.50 and the p value lower than 0.05.

### Cell trajectory analysis

Single-cell trajectories were analyzed using the PAGA algorithm for germ cell populations (Wolf et al., 2019). PAGA delineated differentiation relationships among cell subpopulations based on the cellular atlas. A PAGA network diagram was created: Node size corresponds to the cell count within the subpopulation, while the lines connecting nodes represent inter-subpopulation connections, with line thickness indicating the strength of connectivity. Thicker lines signify more potent connectivity, reflecting stronger differentiation and inheritance relationships among subpopulations.

### Regulon analysis

Regulon analysis was performed using the SCENIC R package. The log-normalized expression matrix generated by Seurat was utilized as the input data. The activity of individual regulons in each single cell was evaluated by computing the area under the curve (AUC) with the AUCell R package.

### FISH assay

FISH examination was carried out using a FISH Kit (RiboBio, China) following the manufacturer’s guidelines. A specific probe designed for Creb3l4 was synthesized by RiboBio. Images were acquired using a Zeiss laser confocal microscope (LSM800, Carl Zeiss).

### Statistical analysis

The data are presented as mean ± SEM and were analyzed using GraphPad Prism nine software. Significance between two groups was assessed with an unpaired Student’s t-test. For multiple groups, a one-way ANOVA was conducted, followed by Dunnett’s test for *post hoc* comparisons. A p-value below 0.05 indicated statistical significance.

## Data Availability

The datasets presented in this study can be found in online repositories. The names of the repository/repositories and accession number(s) can be found in the article/Supplementary Material.

## References

[B1] Adham I. M. Eck T. J. Mierau K. Müller N. Sallam M. A. Paprotta I. (2005). Reduction of spermatogenesis but not fertility in Creb3l4-deficient mice. Mol. Cell. Biol. 25, 7657–7664. 10.1128/MCB.25.17.7657-7664.2005 16107712 PMC1190296

[B2] Butler A. Hoffman P. Smibert P. Papalexi E. Satija R. (2018). Integrating single-cell transcriptomic data across different conditions, technologies, and species. Nat. Biotechnol. 36, 411–420. 10.1038/nbt.4096 29608179 PMC6700744

[B3] Cao L.-H. Qiao J.-Y. Huang H.-Y. Fang X.-Y. Zhang R. Miao M.-S. (2019). PI3K-AKT signaling activation and icariin: the potential effects on the perimenopausal depression-like rat model. Molecules 24, 3700. 10.3390/molecules24203700 31618892 PMC6832648

[B4] Chao H.-H. Zhang Y. Dong P.-Y. Gurunathan S. Zhang X.-F. (2023). Comprehensive review on the positive and negative effects of various important regulators on male spermatogenesis and fertility. Front. Nutr. 9, 1063510. 10.3389/fnut.2022.1063510 36726821 PMC9884832

[B5] Chuang H.-L. Bharath Kumar V. Day C. H. Ho C.-C. Ho T.-J. Chen R.-J. (2021). Epimedium promotes steroidogenesis by CREB activation-mediated mitochondrial fusion in endosulfan treated leydig cells. Environ. Toxicol. 36, 1873–1879. 10.1002/tox.23307 34089567

[B6] Ding Z. Chen X. Tang D. Ye T. Yang J. Yu Y. (2025). Comparisons of the bioavailability of icariin, icariside II, and epimedin C in rats after oral administration of total flavonoids of Epimedium brevicornu maxim and its three formulations. J. Pharm. Biomed. Anal. 255, 116631. 10.1016/j.jpba.2024.116631 39671909

[B7] Fan C. Yang Y. Liu Y. Jiang S. Di S. Hu W. (2016). Icariin displays anticancer activity against human esophageal cancer cells *via* regulating endoplasmic reticulum stress-mediated apoptotic signaling. Sci. Rep. 6, 21145. 10.1038/srep21145 26892033 PMC4759694

[B8] Jaeschke H. Ramachandran A. (2024). Ferroptosis and intrinsic drug-induced liver injury by acetaminophen and other drugs: a critical evaluation and historical perspective. J. Clin. Transl. Hepatol. 12, 1057–1066. 10.14218/JCTH.2024.00324 39649034 PMC11622198

[B9] Jia G. Zhang Y. Li W. Dai H. (2019). Neuroprotective role of icariin in experimental spinal cord injury *via* its antioxidant, anti-neuroinflammatory and anti-apoptotic properties. Mol. Med. Rep. 20, 3433–3439. 10.3892/mmr.2019.10537 31432160

[B10] Jin H. Xue B. Chen X. Ma T. Ma Y. Zou H. (2025). Polystyrene microplastics induced spermatogenesis disorder *via* disrupting mitochondrial function through the regulation of the Sirt1-Pgc1α signaling pathway in male mice. Environ. Pollut. 364, 125364. 10.1016/j.envpol.2024.125364 39577614

[B11] Khodadadi R. Jalali A. Moghadasi S. Farahani M. (2025). Environmental exposure to titanium dioxide nanoparticles disrupts DAZL gene expression and male reproductive function in mice: protective role of lutein. Food Chem. Toxicol. 195, 115128. 10.1016/j.fct.2024.115128 39580016

[B12] Kong L. Liu J. Wang J. Luo Q. Zhang H. Liu B. (2015). Icariin inhibits TNF-α/IFN-γ induced inflammatory response *via* inhibition of the substance P and p38-MAPK signaling pathway in human keratin ocytes. Int. Immunopharmacol. 29, 401–407. 10.1016/j.intimp.2015.10.023 26507164

[B13] Li W. Wang M. Wang L. Ji S. Zhang J. Zhang C. (2014). Icariin synergizes with arsenic trioxide to suppress human hepatocellular carcinoma. Cell. Biochem. Biophys. 68, 427–436. 10.1007/s12013-013-9724-3 23975599

[B14] Li C. Yang S. Ma H. Ruan M. Fang L. Cheng J. (2021). Influence of icariin on inflammation, apoptosis, invasion, and tumor immunity in cervical cancer by reducing the TLR4/MyD88/NF-κB and Wnt/β-catenin pathways. Cancer Cell. Int. 21, 206. 10.1186/s12935-021-01910-2 33849528 PMC8045342

[B15] Li J. Chen D. Suo J. Li J. Zhang Y. Wang Y. (2024a). Triptolide induced spermatogenesis dysfunction *via* ferroptosis activation by promoting K63-linked GPX4 polyubiquitination in spermatocytes. Chem. Biol. Interact. 399, 111130. 10.1016/j.cbi.2024.111130 38960301

[B16] Li X. Shen K. Yuan D. Li X. Quan J. Tian F. (2024b). Sodium arsenite impairs sperm quality *via* downregulating the ZMYND15 and ZMYND10. Environ. Toxicol. 39, 4385–4396. 10.1002/tox.24327 38798119

[B17] Li F. Zhu F. Wang S. Hu H. Zhang D. He Z. (2024c). Icariin alleviates cisplatin-induced premature ovarian failure by inhibiting ferroptosis through activation of the Nrf2/ARE pathway. Sci. Rep. 14, 17318. 10.1038/s41598-024-67557-x 39068256 PMC11283570

[B18] Li S. Ma S. Wang L. Zhan D. Jiang S. Zhang Z. (2024d). ATF3 as a response factor to regulate Cd-induced reproductive damage by activating the NRF2/HO-1 ferroptosis pathway. Ecotoxicol. Environ. Saf. 285, 117114. 10.1016/j.ecoenv.2024.117114 39357374

[B19] Liu Y. Yang X. Gan J. Chen S. Xiao Z.-X. Cao Y. (2022). CB-Dock2: improved protein-ligand blind docking by integrating cavity detection, docking and homologous template fitting. Nucleic Acids Res. 50, W159–W164. 10.1093/nar/gkac394 35609983 PMC9252749

[B20] Lun A. T. L. Riesenfeld S. Andrews T. Dao T. P. Gomes T. J. Marioni J. C. (2019). EmptyDrops: distinguishing cells from empty droplets in droplet-based single-cell RNA sequencing data. Genome Biol. 20, 63. 10.1186/s13059-019-1662-y 30902100 PMC6431044

[B21] Ma B. Qi H. Li J. Xu H. Chi B. Zhu J. (2015). Triptolide disrupts fatty acids and peroxisome proliferator-activated receptor (PPAR) levels in male mice testes followed by testicular injury: a GC-MS based metabolomics study. Toxicology 336, 84–95. 10.1016/j.tox.2015.07.008 26219505

[B22] Ma B. Zhang J. Zhu Z. Zhao A. Zhou Y. Ying H. (2019). Luteolin ameliorates testis injury and blood-testis barrier disruption through the Nrf2 signaling pathway and by upregulating Cx43. Mol. Nutr. Food Res. 63, e1800843. 10.1002/mnfr.201800843 30924608

[B23] McGinnis C. S. Murrow L. M. Gartner Z. J. (2019). DoubletFinder: doublet detection in single-cell RNA sequencing data using artificial nearest neighbors. Cell. Syst. 8, 329–337.e324. 10.1016/j.cels.2019.03.003 30954475 PMC6853612

[B24] Mok S.-K. Chen W.-F. Lai W.-P. Leung P.-C. Wang X.-L. Yao X.-S. (2010). Icariin protects against bone loss induced by oestrogen deficiency and activates oestrogen receptor-dependent osteoblastic functions in UMR 106 cells. Br. J. Pharmacol. 159, 939–949. 10.1111/j.1476-5381.2009.00593.x 20128811 PMC2829219

[B25] Ni G. Zhang X. Afedo S. Y. Rui R. (2020). Evaluation of the protective effects of icariin on nicotine-induced reproductive toxicity in male mouse -a pilot study. Reprod. Biol. Endocrinol. 18, 65. 10.1186/s12958-020-00620-0 32552695 PMC7302363

[B26] Pohl E. Gromoll J. Wistuba J. Laurentino S. (2021). Healthy ageing and spermatogenesis. Reproduction 161, R89–R101. 10.1530/rep-20-0633 33574214

[B27] Sánchez-Gutiérrez M. Izquierdo-Vega A. J. Madrigal-Santillán E. O. Velázquez-González C. Izquierdo-Vega J. A. (2024). Icariin as a treatment proposal in mammalian reproduction. Pharm. (Basel) 17, 1104. 10.3390/ph17091104 39338269 PMC11434857

[B28] Segal T. R. Giudice L. C. (2019). Before the beginning: environmental exposures and reproductive and obstetrical outcomes. Fertil. Steril. 112, 613–621. 10.1016/j.fertnstert.2019.08.001 31561863

[B29] Senior A. W. Evans R. Jumper J. Kirkpatrick J. Sifre L. Green T. (2020). Improved protein structure prediction using potentials from deep learning. Nature 577, 706–710. 10.1038/s41586-019-1923-7 31942072

[B30] Sharma A. Minhas S. Dhillo W. S. Jayasena C. N. (2021). Male infertility due to testicular disorders. J. Clin. Endocrinol. Metab. 106, e442–e459. 10.1210/clinem/dgaa781 33295608 PMC7823320

[B31] Stockwell B. R. Friedmann Angeli J. P. Bayir H. Bush A. I. Conrad M. Dixon S. J. (2017). Ferroptosis: a regulated cell death nexus linking metabolism, redox biology, and disease. Cell. 171, 273–285. 10.1016/j.cell.2017.09.021 28985560 PMC5685180

[B32] Sun J. Wang D. Lin J. Liu Y. Xu L. Lv R. (2019). Icariin protects mouse leydig cell testosterone synthesis from the adverse effects of di(2-ethylhexyl) phthalate. Toxicol. Appl. Pharmacol. 378, 114612. 10.1016/j.taap.2019.114612 31175881

[B33] Velpula K. K. Rehman A. A. Chigurupati S. Sanam R. Inampudi K. K. Akila C. S. (2012). Computational analysis of human and mouse CREB3L4 protein. Bioinformation 8, 574–577. 10.6026/97320630008574 22829733 PMC3398783

[B34] Wan J. Ren H. Wang J. (2019). Iron toxicity, lipid peroxidation and ferroptosis after intracerebral haemorrhage. Stroke Vasc. Neurol. 4, 93–95. 10.1136/svn-2018-000205 31338218 PMC6613877

[B35] Wang Z. Wang D. Yang D. Zhen W. Zhang J. Peng S. (2018). The effect of icariin on bone metabolism and its potential clinical application. Osteoporos. Int. 29, 535–544. 10.1007/s00198-017-4255-1 29110063

[B36] Wang S. Ma J. Zeng Y. Zhou G. Wang Y. Zhou W. (2021). Icariin, an up-and-coming bioactive compound against neurological diseases: network pharmacology-based study and literature review. Drug Des. Devel Ther. 15, 3619–3641. 10.2147/DDDT.S310686 34447243 PMC8384151

[B37] Wang Z. Zhou Z. Zhang L. Li X. Li M. Pan Y. (2023). Efficacy and safety of nonpharmacological strategies for the treatment of oligoasthenospermia: a systematic review and Bayesian network meta-analysis. Eur. J. Med. Res. 28, 6. 10.1186/s40001-022-00968-6 36600309 PMC9811722

[B38] Wang Y. Chembazhi U. V. Yee D. Chen S. Ji J. Wang Y. (2024). PTBP1 mediates sertoli cell actin cytoskeleton organization by regulating alternative splicing of actin regulators. Nucleic Acids Res. 52, 12244–12261. 10.1093/nar/gkae862 39373517 PMC11551747

[B39] Wolf F. A. Hamey F. K. Plass M. Solana J. Dahlin J. S. Göttgens B. (2019). PAGA: graph abstraction reconciles clustering with trajectory inference through atopology preserving map of single cells. Genome Biol. 20, 59. 10.1186/s13059-019-1663-x 30890159 PMC6425583

[B40] Xi C. Peng S. Wu Z. Zhou Q. Zhou J. (2017). Toxicity of triptolide and the molecular mechanisms involved. Biomed. Pharmacother. 90, 531–541. 10.1016/j.biopha.2017.04.003 28402922

[B41] Xu C.-Q. Liu B.-J. Wu J.-F. Xu Y.-C. Duan X.-H. Cao Y.-X. (2010). Icariin attenuates LPS-induced acute inflammatory responses: involvement of PI3K/Akt and NF-kappaB signaling pathway. Eur. J. Pharmacol. 642, 146–153. 10.1016/j.ejphar.2010.05.012 20519138

[B42] Xu C. Huang X. Tong Y. Feng X. Wang Y. Wang C. (2020). Icariin modulates the sirtuin/NF-κB pathway and exerts anti-aging effects in human lung fibroblasts. Mol. Med. Rep. 22, 3833–3839. 10.3892/mmr.2020.11458 33000191 PMC7533484

[B43] Yang X. He L. Li X. Wang L. Bu T. Yun D. (2024). Triptolide exposure triggers testicular vacuolization injury by disrupting the sertoli cell junction and cytoskeletal organization *via* the AKT/mTOR signaling pathway. Ecotoxicol. Environ. Saf. 279, 116502. 10.1016/j.ecoenv.2024.116502 38788563

[B44] Zangene S. Morovvati H. Anbara H. Hye Khan M. A. Goorani S. (2024). Polystyrene microplastics cause reproductive toxicity in male mice. Food Chem. Toxicol. 194, 115083. 10.1016/j.fct.2024.115083 39521238

[B45] Zeng Y. Yang Q. Ouyang Y. Lou Y. Cui H. Deng H. (2023). Nickel induces blood-testis barrier damage through ROS-mediated p38 MAPK pathways in mice. Redox Biol. 67, 102886. 10.1016/j.redox.2023.102886 37742495 PMC10520947

[B46] Zhang X. Sun H. Su Q. Lin T. Zhang H. Zhang J. (2017). Antidepressant-like activity of icariin mediated by group I mGluRs in prenatally stressed offspring. Brain Dev. 39, 593–600. 10.1016/j.braindev.2017.03.021 28395974

[B47] Zhang Q. Zhang Z. Liu Z. Wang C. Chen H. Shen L. (2024). Deficiency in the Rab25 gene leads to a decline in male fertility and testicular injury: impact on the regulation of germ cell proliferation and apoptosis. Exp. Cell. Res. 442, 114285. 10.1016/j.yexcr.2024.114285 39424096

[B48] Zhou H. Yuan Y. Liu Y. Deng W. Zong J. Bian Z.-Y. (2014). Icariin attenuates angiotensin II-induced hypertrophy and apoptosis in H9c2 cardiomyocytes by inhibiting reactive oxygen species-dependent JNK and p38 pathways. Exp. Ther. Med. 7, 1116–1122. 10.3892/etm.2014.1598 24940396 PMC3991546

[B49] Zhou Z. Luo J. Wang J. Li L. Kong L. (2015). Simultaneous enrichment and separation of flavonoids from herba epimed ii by macroporous resins coupled with preparative chromatographic method. Nat. Prod. Res. 29, 185–188. 10.1080/14786419.2014.964704 25277166

[B50] Zhu H. Wang H. Cheng Y. Liu D. Zhang A. Wen Z. (2022). Hadh deficiency induced oligoasthenoteratozoospermia through the TNF-α/Bcl-2 pathway in male mice. FASEB J. 36, e22661. 10.1096/fj.202201144r 36398584

