# Supplementary figures and images for: Icariin alleviates triptolide-induced testicular vacuolization via modulating germline ferroptosis and blood-testis barrier integrity
Source: Front Cell Dev Biol. 2026 Jul 2;14:1846734. doi: 10.3389/fcell.2026.1846734 (PMC13372769; doi:10.3389/fcell.2026.1846734)

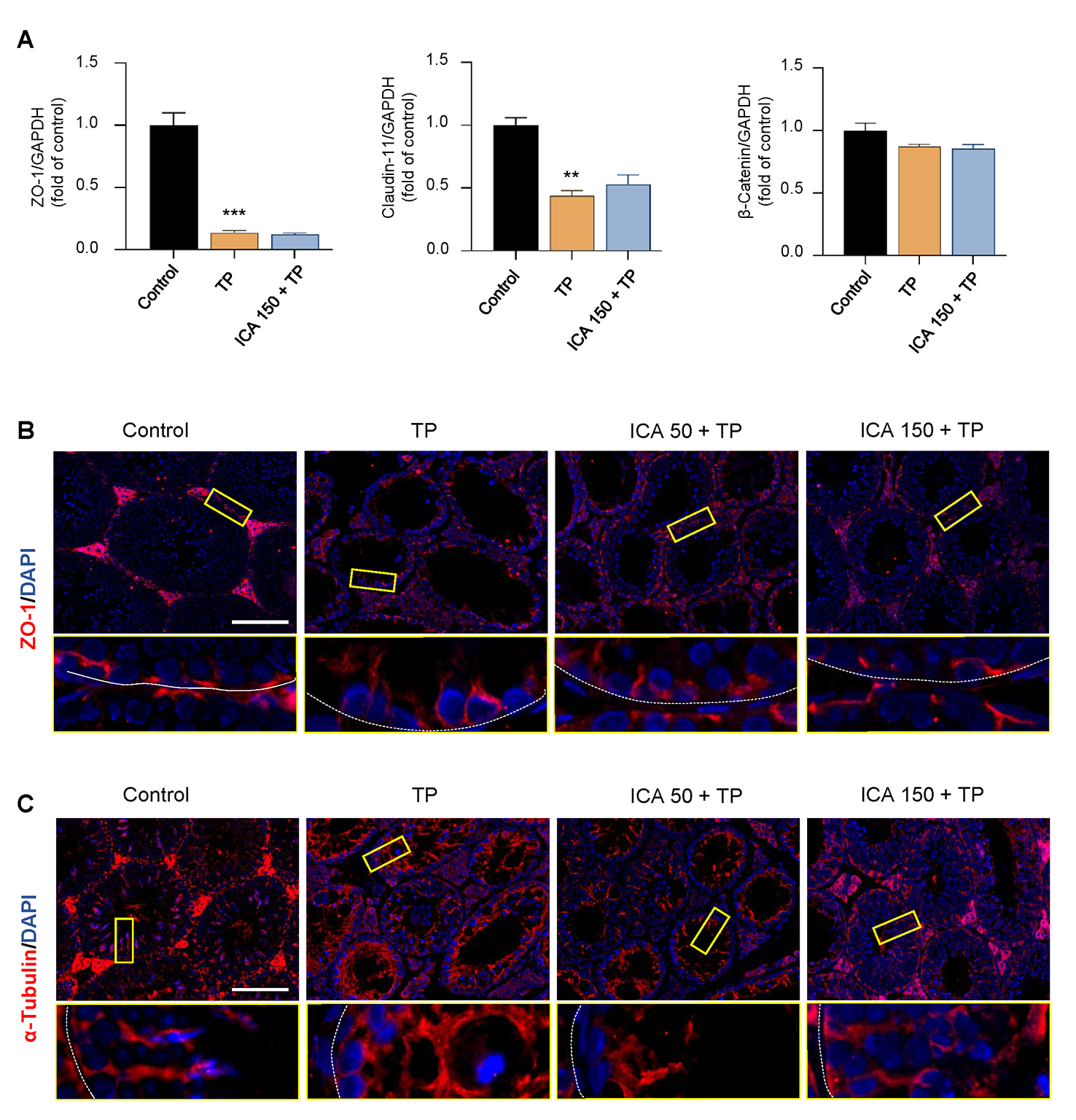

Supplement: Supplementary file 1 [file Image3.TIF]

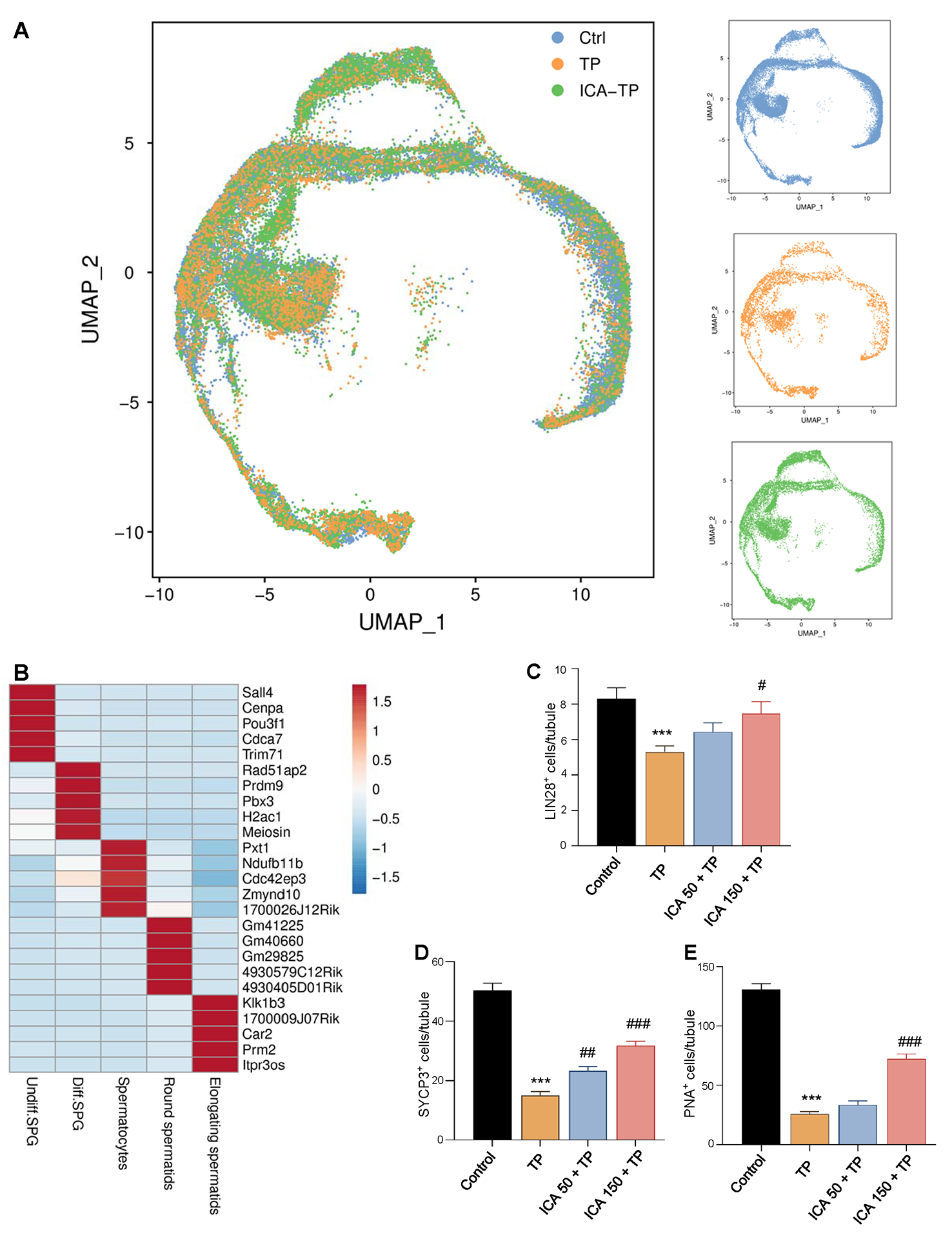

Supplement: Supplementary file 2 [file Image4.TIF]

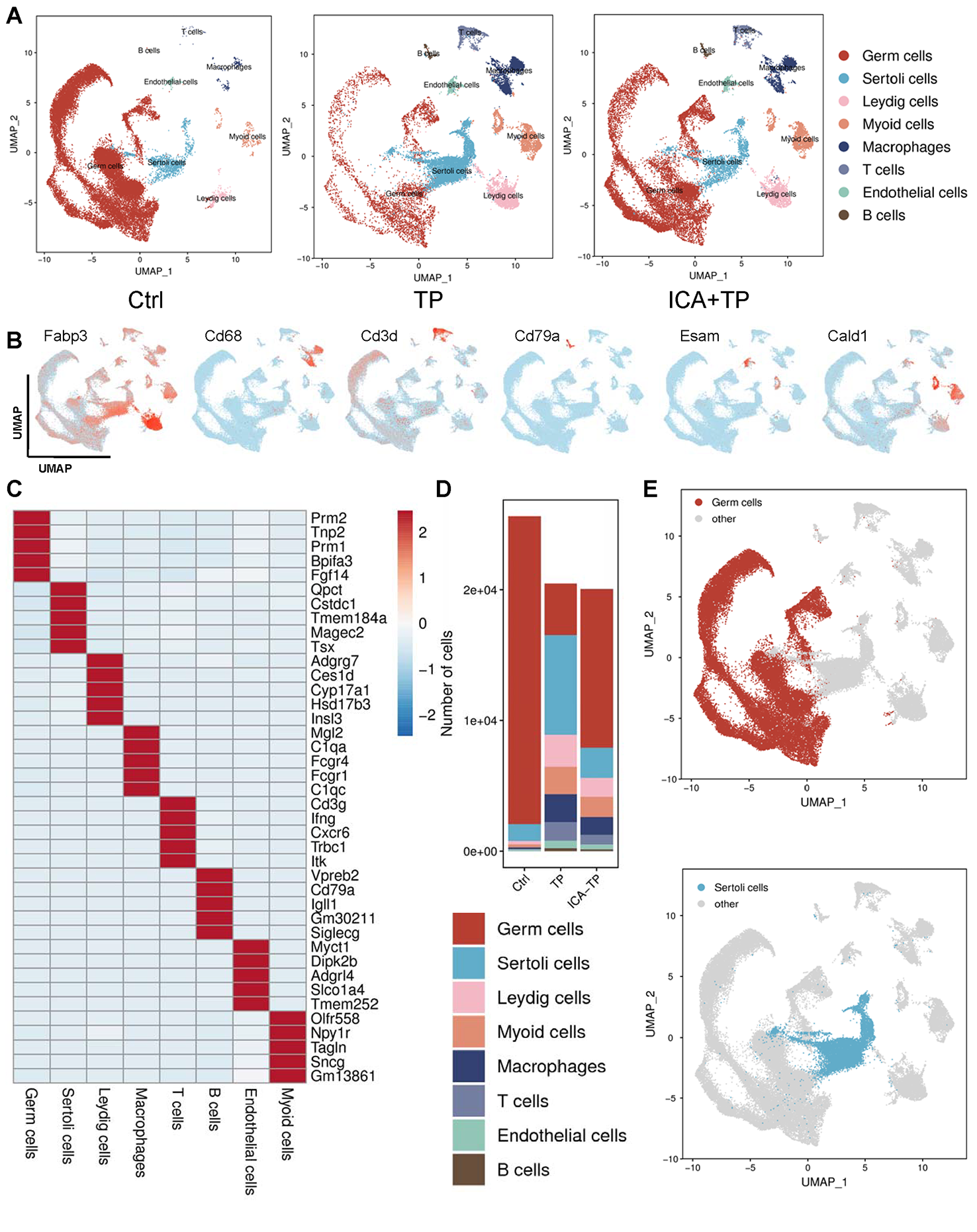

Supplement: Supplementary file 3 [file Image2.TIF]

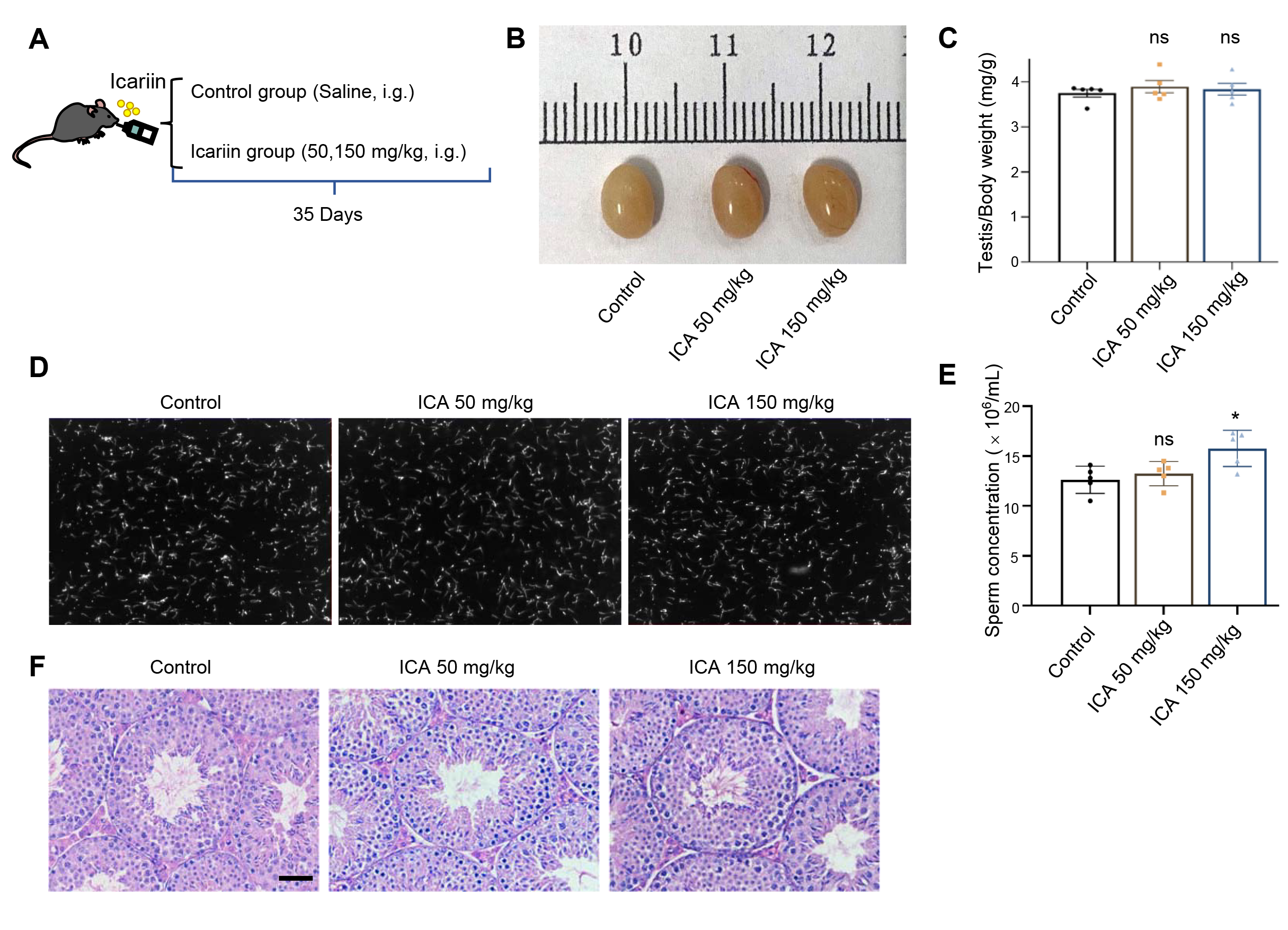

Supplement: Supplementary file 4 [file Image1.TIF]

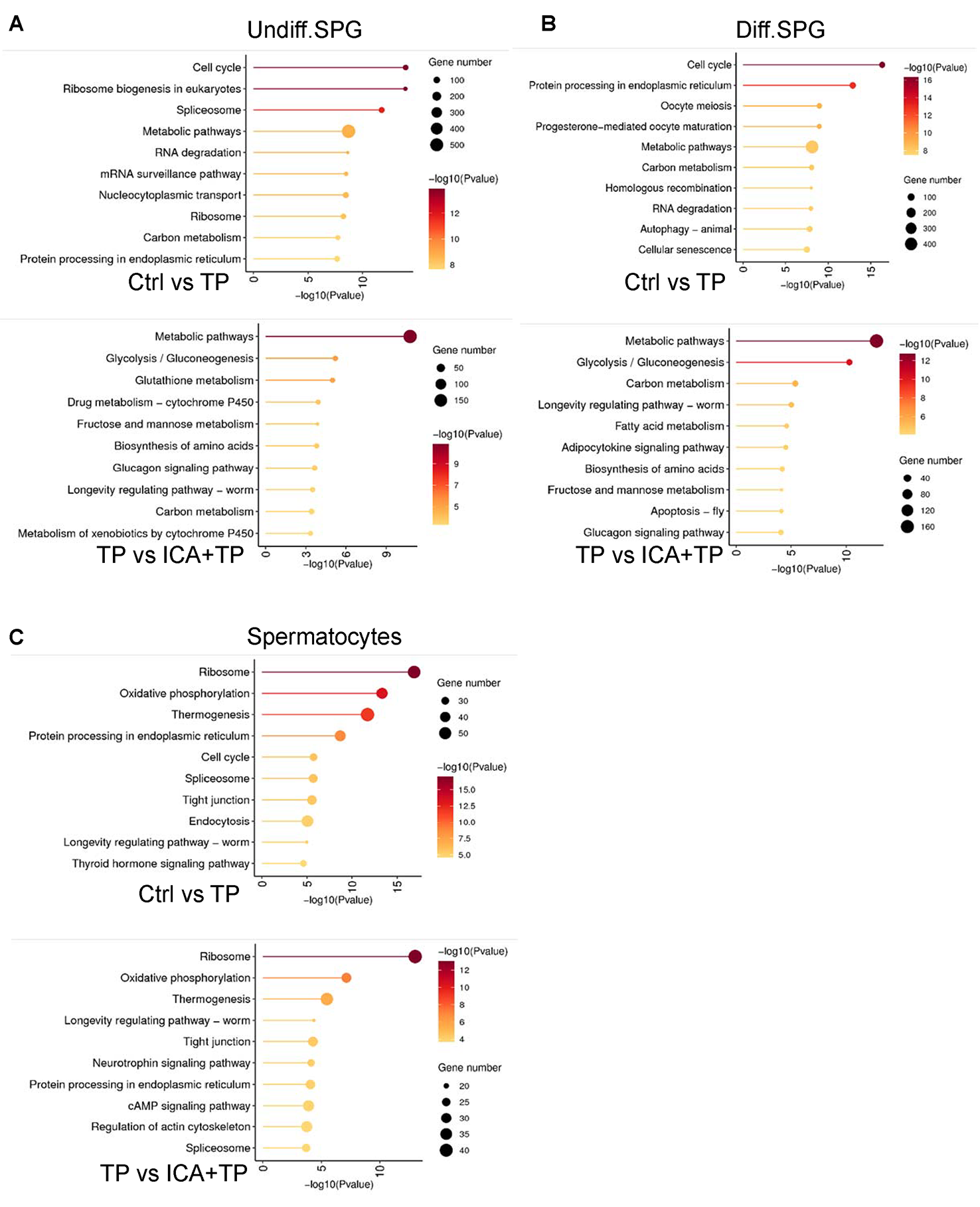

Supplement: Supplementary file 10 [file Image5.TIF]
