# Supplementary material for: Icariin alleviates triptolide-induced testicular vacuolization via modulating germline ferroptosis and blood-testis barrier integrity
Source: Front Cell Dev Biol. 2026 Jul 2;14:1846734. doi: 10.3389/fcell.2026.1846734 (PMC13372769; doi:10.3389/fcell.2026.1846734)
Supplement: Supplementary file 5 [file Table2.docx]

**Table S2. Detailed residues for GPX4/Triptolide interactions.**

| **Receptor** | **Atom index** | **Residue name** | **Residue position** | **Atom name** | **Ligand** | **Atom index** | **Atom name** | **Distance** |
| --- | --- | --- | --- | --- | --- | --- | --- | --- |
| **GPX4** | 73 | ILE | 9 | CG2 | **Triptolide** | 3 | C | 3.49 |
| **GPX4** | 700 | MET | 89 | CG | **Triptolide** | 6 | C | 3.79 |
| **GPX4** | 699 | MET | 89 | CB | **Triptolide** | 6 | C | 3.82 |
| **GPX4** | 604 | LYS | 77 | CE | **Triptolide** | 6 | C | 3.77 |
| **GPX4** | 602 | LYS | 77 | CG | **Triptolide** | 6 | C | 3.84 |
| **GPX4** | 82 | ASP | 10 | OD2 | **Triptolide** | 11 | C | 3.68 |
| **GPX4** | 73 | ILE | 9 | CG2 | **Triptolide** | 11 | C | 3.92 |
| **GPX4** | 693 | ASP | 88 | OD1 | **Triptolide** | 12 | C | 3.46 |
| **GPX4** | 82 | ASP | 10 | OD2 | **Triptolide** | 13 | C | 3.91 |
| **GPX4** | 64 | ASP | 8 | CG | **Triptolide** | 14 | C | 3.84 |
| **GPX4** | 82 | ASP | 10 | OD2 | **Triptolide** | 14 | C | 3.58 |
| **GPX4** | 66 | ASP | 8 | OD2 | **Triptolide** | 14 | C | 3.31 |
| **GPX4** | 82 | ASP | 10 | OD2 | **Triptolide** | 15 | C | 3.58 |
| **GPX4** | 665 | VAL | 85 | CG1 | **Triptolide** | 18 | C | 3.79 |
| **GPX4** | 663 | VAL | 85 | O | **Triptolide** | 18 | C | 3.2 |
| **GPX4** | 662 | VAL | 85 | C | **Triptolide** | 18 | C | 4.0 |
| **GPX4** | 664 | VAL | 85 | CB | **Triptolide** | 18 | C | 3.85 |
| **GPX4** | 663 | VAL | 85 | O | **Triptolide** | 19 | C | 3.35 |
| **GPX4** | 635 | ALA | 81 | CB | **Triptolide** | 19 | C | 3.69 |
| **GPX4** | 700 | MET | 89 | CG | **Triptolide** | 20 | C | 3.7 |
| **GPX4** | 664 | VAL | 85 | CB | **Triptolide** | 20 | C | 3.85 |
| **GPX4** | 630 | ALA | 80 | CB | **Triptolide** | 20 | C | 3.3 |
| **GPX4** | 695 | MET | 89 | N | **Triptolide** | 21 | O | 3.26 |
| **GPX4** | 688 | ASP | 88 | CA | **Triptolide** | 21 | O | 3.85 |
| **GPX4** | 679 | PHE | 87 | O | **Triptolide** | 21 | O | 3.3 |
| **GPX4** | 604 | LYS | 77 | CE | **Triptolide** | 23 | O | 2.96 |
| **GPX4** | 603 | LYS | 77 | CD | **Triptolide** | 23 | O | 3.75 |
| **GPX4** | 602 | LYS | 77 | CG | **Triptolide** | 23 | O | 3.35 |
| **GPX4** | 94 | HIS | 12 | ND1 | **Triptolide** | 25 | O | 3.69 |
| **GPX4** | 66 | ASP | 8 | OD2 | **Triptolide** | 25 | O | 3.43 |
| **GPX4** | 670 | LYS | 86 | O | **Triptolide** | 26 | O | 3.93 |
| **GPX4** | 678 | PHE | 87 | C | **Triptolide** | 26 | O | 3.98 |
| **GPX4** | 669 | LYS | 86 | C | **Triptolide** | 26 | O | 3.44 |
| **GPX4** | 676 | PHE | 87 | N | **Triptolide** | 26 | O | 3.69 |
| **GPX4** | 668 | LYS | 86 | CA | **Triptolide** | 26 | O | 3.41 |
| **GPX4** | 667 | LYS | 86 | N | **Triptolide** | 26 | O | 3.73 |
| **GPX4** | 679 | PHE | 87 | O | **Triptolide** | 27 | H | 2.2 |

**Note: This table extracted interactions within a 4 Ångstrom range.**
