# Supplementary material for: Icariin alleviates triptolide-induced testicular vacuolization via modulating germline ferroptosis and blood-testis barrier integrity
Source: Front Cell Dev Biol. 2026 Jul 2;14:1846734. doi: 10.3389/fcell.2026.1846734 (PMC13372769; doi:10.3389/fcell.2026.1846734)
Supplement: Supplementary file 7 [file Table3.docx]

**Table S3. Detailed residues for GPX4/Icariin interactions.**

| **Receptor** | **Atom index** | **Residue name** | **Residue position** | **Atom name** | **Ligand** | **Atom index** | **Atom name** | **Distance** |
| --- | --- | --- | --- | --- | --- | --- | --- | --- |
| **GPX4** | 693 | ASP | 88 | OD1 | **Icariin** | 2 | C | 3.52 |
| **GPX4** | 693 | ASP | 88 | OD1 | **Icariin** | 4 | C | 3.43 |
| **GPX4** | 693 | ASP | 88 | OD1 | **Icariin** | 5 | C | 3.35 |
| **GPX4** | 695 | MET | 89 | N | **Icariin** | 6 | C | 3.86 |
| **GPX4** | 670 | LYS | 86 | O | **Icariin** | 12 | C | 3.39 |
| **GPX4** | 669 | LYS | 86 | C | **Icariin** | 12 | C | 3.89 |
| **GPX4** | 668 | LYS | 86 | CA | **Icariin** | 12 | C | 3.97 |
| **GPX4** | 673 | LYS | 86 | CD | **Icariin** | 14 | C | 3.58 |
| **GPX4** | 673 | LYS | 86 | CD | **Icariin** | 15 | C | 3.82 |
| **GPX4** | 663 | VAL | 85 | O | **Icariin** | 22 | C | 2.98 |
| **GPX4** | 662 | VAL | 85 | C | **Icariin** | 22 | C | 3.85 |
| **GPX4** | 664 | VAL | 85 | CB | **Icariin** | 22 | C | 3.88 |
| **GPX4** | 663 | VAL | 85 | O | **Icariin** | 23 | C | 3.59 |
| **GPX4** | 700 | MET | 89 | CG | **Icariin** | 24 | C | 3.85 |
| **GPX4** | 604 | LYS | 77 | CE | **Icariin** | 26 | C | 3.98 |
| **GPX4** | 66 | ASP | 8 | OD2 | **Icariin** | 28 | C | 3.78 |
| **GPX4** | 66 | ASP | 8 | OD2 | **Icariin** | 29 | C | 3.11 |
| **GPX4** | 111 | VAL | 14 | CG2 | **Icariin** | 30 | C | 3.68 |
| **GPX4** | 66 | ASP | 8 | OD2 | **Icariin** | 30 | C | 3.84 |
| **GPX4** | 66 | ASP | 8 | OD2 | **Icariin** | 31 | C | 3.74 |
| **GPX4** | 66 | ASP | 8 | OD2 | **Icariin** | 33 | C | 3.9 |
| **GPX4** | 696 | MET | 89 | CA | **Icariin** | 35 | O | 3.73 |
| **GPX4** | 695 | MET | 89 | N | **Icariin** | 35 | O | 2.96 |
| **GPX4** | 699 | MET | 89 | CB | **Icariin** | 35 | O | 3.53 |
| **GPX4** | 673 | LYS | 86 | CD | **Icariin** | 36 | O | 3.6 |
| **GPX4** | 696 | MET | 89 | CA | **Icariin** | 37 | O | 3.95 |
| **GPX4** | 695 | MET | 89 | N | **Icariin** | 37 | O | 3.27 |
| **GPX4** | 63 | ASP | 8 | CB | **Icariin** | 37 | O | 3.74 |
| **GPX4** | 698 | MET | 89 | O | **Icariin** | 37 | O | 2.62 |
| **GPX4** | 697 | MET | 89 | C | **Icariin** | 37 | O | 3.59 |
| **GPX4** | 679 | PHE | 87 | O | **Icariin** | 38 | O | 3.58 |
| **GPX4** | 604 | LYS | 77 | CE | **Icariin** | 39 | O | 3.28 |
| **GPX4** | 664 | VAL | 85 | CB | **Icariin** | 40 | O | 3.18 |
| **GPX4** | 630 | ALA | 80 | CB | **Icariin** | 40 | O | 3.56 |
| **GPX4** | 629 | ALA | 80 | O | **Icariin** | 40 | O | 3.66 |
| **GPX4** | 631 | ALA | 81 | N | **Icariin** | 40 | O | 3.35 |
| **GPX4** | 628 | ALA | 80 | C | **Icariin** | 40 | O | 3.42 |
| **GPX4** | 600 | LYS | 77 | O | **Icariin** | 40 | O | 3.82 |
| **GPX4** | 663 | VAL | 85 | O | **Icariin** | 41 | O | 3.14 |
| **GPX4** | 632 | ALA | 81 | CA | **Icariin** | 41 | O | 3.78 |
| **GPX4** | 604 | LYS | 77 | CE | **Icariin** | 42 | O | 3.7 |
| **GPX4** | 603 | LYS | 77 | CD | **Icariin** | 42 | O | 3.85 |
| **GPX4** | 602 | LYS | 77 | CG | **Icariin** | 42 | O | 2.84 |
| **GPX4** | 601 | LYS | 77 | CB | **Icariin** | 42 | O | 3.9 |
| **GPX4** | 598 | LYS | 77 | CA | **Icariin** | 42 | O | 3.92 |
| **GPX4** | 142 | LYS | 18 | NZ | **Icariin** | 43 | O | 3.74 |
| **GPX4** | 66 | ASP | 8 | OD2 | **Icariin** | 44 | O | 3.66 |
| **GPX4** | 111 | VAL | 14 | CG2 | **Icariin** | 47 | O | 3.56 |
| **GPX4** | 91 | HIS | 12 | CB | **Icariin** | 47 | O | 3.55 |
| **GPX4** | 66 | ASP | 8 | OD2 | **Icariin** | 47 | O | 3.78 |
| **GPX4** | 94 | HIS | 12 | ND1 | **Icariin** | 48 | O | 3.02 |
| **GPX4** | 64 | ASP | 8 | CG | **Icariin** | 48 | O | 3.79 |
| **GPX4** | 79 | ASP | 10 | CB | **Icariin** | 48 | O | 3.74 |
| **GPX4** | 63 | ASP | 8 | CB | **Icariin** | 49 | H | 3.46 |
| **GPX4** | 700 | MET | 89 | CG | **Icariin** | 50 | H | 3.93 |
| **GPX4** | 664 | VAL | 85 | CB | **Icariin** | 50 | H | 3.32 |
| **GPX4** | 630 | ALA | 80 | CB | **Icariin** | 52 | H | 3.9 |
| **GPX4** | 602 | LYS | 77 | CG | **Icariin** | 52 | H | 3.12 |
| **GPX4** | 598 | LYS | 77 | CA | **Icariin** | 52 | H | 3.54 |
| **GPX4** | 91 | HIS | 12 | CB | **Icariin** | 55 | H | 2.78 |
| **GPX4** | 94 | HIS | 12 | ND1 | **Icariin** | 55 | H | 3.67 |
| **GPX4** | 92 | HIS | 12 | CG | **Icariin** | 55 | H | 3.3 |
| **GPX4** | 66 | ASP | 8 | OD2 | **Icariin** | 55 | H | 3.64 |
| **GPX4** | 96 | HIS | 12 | NE2 | **Icariin** | 56 | H | 3.81 |
| **GPX4** | 95 | HIS | 12 | CE1 | **Icariin** | 56 | H | 2.52 |
| **GPX4** | 79 | ASP | 10 | CB | **Icariin** | 56 | H | 3.4 |

**Note: This table extracted interactions within a 4 Ångstrom range.**
