# Supplementary material for: Icariin alleviates triptolide-induced testicular vacuolization via modulating germline ferroptosis and blood-testis barrier integrity
Source: Front Cell Dev Biol. 2026 Jul 2;14:1846734. doi: 10.3389/fcell.2026.1846734 (PMC13372769; doi:10.3389/fcell.2026.1846734)
Supplement: Supplementary file 9 [file Table6.docx]

**Table S6. Detailed information for antibodies used in this study.**

| **Antigen** | **Source** | **Company** | **Application** | **Dilution** | **Cat No.** |
| --- | --- | --- | --- | --- | --- |
| LIN28 | Rabbit | Abcam | IF | 1: 200 | ab46020 |
| SYCP3 | Mouse | Santa Cruz | IF | 1: 20 | sc-74569 |
| DDX4 | Rabbit | Abcam | IF | 1: 200 | ab13840 |
| WT1 | Rabbit | Abcam | IF | 1: 100 | ab89901 |
| ZO-1 | Rabbit | Proteintech | WB | 1:500 | 21773-1-AP |
| ZO-1 | Mouse | Invitrogen | IF | 1: 100 | 33-9100 |
| β-Catenin | Rabbit | Invitrogen | WB | 1: 500 | 71-2700 |
| Claudin-11 | Rabbit | Invitrogen | WB | 1: 500 | 36-4500 |
| Vimentin | Rabbit | Cell Signaling Technology | IF; WB | 1: 50 (IF)  1:500 (WB) | 5741S |
| α-Tubulin | Mouse | Abcam | IF | 1: 100 | ab7291 |
| GPX4 | Mouse | Proteintech | IF | 1:200 | 67763-1-Ig |
| GAPDH | Rabbit | Proteintech | WB | 1:3000 | 60004-1-Ig |
